# Incidence and Predictors of Early and Late Radial Artery Occlusion after Percutaneous Coronary Intervention and Coronary Angiography: A Systematic Review and Meta-Analysis

**DOI:** 10.3390/jcm13195882

**Published:** 2024-10-02

**Authors:** Aisha Khalid, Hans Mautong, Kayode Ahmed, Zaina Aloul, Jose Montero-Cabezas, Silvana Marasco

**Affiliations:** 1 Department of Postgraduate Medical Education, Harvard University, Cambridge, MA 02138, USA; 2 School of Health, Universidad Espíritu Santo-Ecuador, Samborondón 092301, Guayas, Ecuador; 3 Department of Genitourinary Medical Oncology, The University of Texas MD Anderson Cancer Center, Houston, TX 77030, USA; 4 School of Medicine, Cardiff University, Cardiff CF14 4YS, UK; 5 Department of Cardiology, Leiden University Medical Center, 2333 ZA Leiden, The Netherlands; 6 Department of Cardiothoracic Surgery, The Alfred Health, Melbourne, VIC 3004, Australia

**Keywords:** radial artery occlusion, low-molecular-weight heparin (LMWH), oral anticoagulants, percutaneous coronary angiography, coronary angiographic procedures, incidence, predictors

## Abstract

**Introduction:** Trans-radial access for coronary angiography and percutaneous coronary intervention (PCI) has gained popularity due to its advantages over the traditional transfemoral approach. However, radial artery occlusion (RAO) remains a common complication following trans-radial procedures. This study aimed to investigate the incidence of early and late RAO along with their risk factors. **Methods:** Six databases, Medline (Ovid), National Library of Medicine (MeSH), Cochrane Database of Systematic Reviews (Wiley), Embase, Scopus, and Global Index Medicus, were searched. The systematic review and meta-analysis followed the Preferred Reporting Items for Systematic Reviews and Meta-Analyses (PRISMA) guidelines. Data were extracted and analyzed. Using a random-effect model, the primary endpoint was the overall incidence of RAO after invasive coronary procedures. Subgroup analysis and meta-regression were also performed to identify possible predictors of RAO. **Results:** A total of 41 studies with 30,020 patients were included. The overall incidence of RAO was 13% (95% CI = 0.09–0.16). The incidence of early RAO (within 24 h) was 14% (95% CI = 0.10–0.18) in 26 studies, while the incidence of late RAO (after 24 h) was 10% (95% CI = 0.04–0.16) in 22 studies. The average incidence rates of early RAO in studies with catheter sizes of <6 Fr, 6 Fr, and >6 Fr were 9.8%, 9.4%, and 8.8%. The overall effect size of female gender as a predictor was 0.22 with a 95% CI of 0.00–0.44. Age was a potential predictor of early RAO (B = 0.000357; 95% CI = −0.015–0.0027, *p*: 0.006). **Conclusions:** This meta-analysis provides essential information on the incidence of early (14%) and late (10%) RAO following angiographic procedures. Additionally, our findings suggest that female sex and age are possible predictors of RAO. A larger catheter, especially (6 Fr) and hemostatic compression time <90 min post-procedure, substantially reduced the incidence of RAO. The use of oral anticoagulation and the appropriate dosage of low-molecular-weight heparin (LMWH) does reduce RAO, but a comparison between them showed no statistical significance.

## 1. Introduction

Coronary angiography and percutaneous coronary intervention (PCI) are widely used diagnostic and therapeutic procedures for patients with coronary artery disease. Trans-radial access has gained popularity in recent years due to its advantages over the traditional transfemoral approach, including lower bleeding risk, earlier ambulation, and improved patient comfort [1]. However, radial artery occlusion (RAO) remains a common complication following trans-radial procedures, which can limit future use of the same artery for coronary procedures, coronary artery bypass grafting (CABG) [2], or hemodialysis access [3]. The incidence of RAO varies widely in the literature, and the risk factors associated with its occurrence are not well established.

Previous studies have suggested that certain patient and procedural factors, such as female sex, small artery diameter, multiple catheterizations, prolonged radial sheath insertion time, use of high-pressure contrast injections, and prolonged use of hemostatic devices, may increase the risk of RAO [4,5,6]. However, the evidence needs to be more consistent, and additional research is needed to establish the validity of these predictors. To provide a comprehensive understanding of the frequency and predictors of RAO after invasive coronary procedures, we conducted a systematic review and meta-analysis of published studies. Our review includes both early and late RAO, defined as occlusion occurring within 24 h and after 24 h, respectively, following the procedure.

## 2. Methods

### 2.1. Search Strategy

In accordance with the Evidence-Based Guidelines Task Force, a systematic search was conducted in several databases, Medline (Ovid), National Library of Medicine (MeSH), Cochrane Database of Systematic Reviews (Wiley), Embase (Ovid), Scopus, and Global Index Medicus, to identify relevant publications on the topic. The search was conducted by AK and ZA, and the last search was performed on 4 October 2022. The search criteria included no language restrictions, but only articles published in the last decade (between 2012 and 2022) were considered. All references were managed using the reference management software Zotero (Corporation for Digital Scholarship, Vienna, Virginia, USA), and duplicate publications were removed before screening using the systematic review software Rayyan (Cambridge, MA, USA). MeSH terms were used to refine the search, including “radial artery”, “transradial”, “radial access”, “radial artery occlusion”, “radial artery thrombosis”, “oral anticoagulation”, “LMWH”, “percutaneous coronary intervention”, “coronary angiography”, “catheter size”, and “Doppler.” The search criteria were combined using Boolean operators, with the final search string being (1 AND 2 AND 3 AND 4).

### 2.2. Selection Criteria

**Figure 1:**
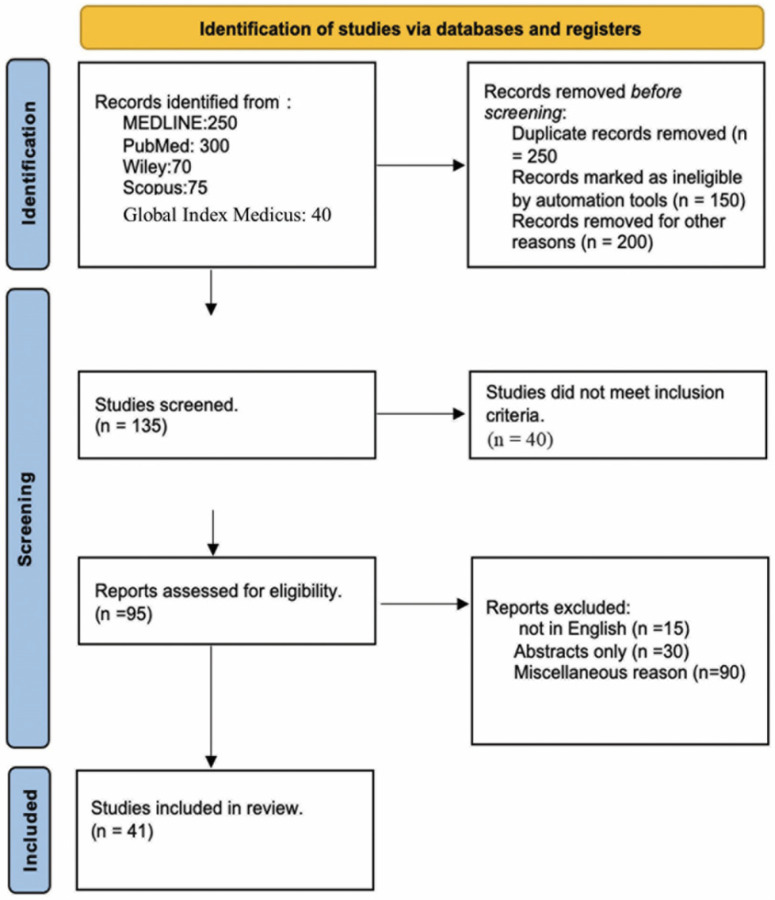
PRISMA—selected studies for the meta-analysis [6].

This systematic review and meta-analysis followed the Preferred Reporting Items for Systematic Reviews and Meta-Analyses (PRISMA) guidelines [7] (Figure 1). The inclusion criteria were studies that provided extractable data on participants and evaluation of RAO rates, as well as studies that discussed access site puncture, use of Doppler ultrasound, sheath and catheter type and size, patient hemostasis strategy and hemostatic band duration, pharmacological and non-pharmacological management, including use of oral anticoagulants, and clinical risk factors for RAO. The exclusion criteria included studies that did not report RAO rates, had irretrievable data, were not in English, presented data only in abstract form, were duplicates, and expert opinions, letters, and editorial studies.

### 2.3. Data Extraction

AK and ZA conducted data extraction, while KA acted as a verifier to ensure the accuracy of the data. Relevant information was extracted from the studies, including study type, author, country, participant demographics, the incidence of RAO (early and late), procedural details, anticoagulation dosage, hemostasis method, methods used to assess radial artery patency, catheter size, predictors of RAO such as age and gender, follow-up duration, and study outcomes. The extracted data were compiled and are presented in Supplemental Tables S1 and S2.

### 2.4. Quality Assessment

A critical appraisal of the 41 selected publications was performed according to the study type. Risk-of-bias assessment of all RCTs was performed by AK and KA using the RoB 2 Cochrane tool by dividing them into low-risk, some concerns, and high-risk types. At the same time, all observational and case–control studies were assessed using a quality checklist from the Newcastle–Ottawa Scale [8] by AK and KA and dividing the studies into low and high quality. The scores were measured using a standardized star allocation method for the sample prototype, and outcomes are shown in Supplemental Tables S3 and S4.

### 2.5. Outcomes

The primary endpoint of this meta-analysis was the overall incidence of RAO after invasive coronary procedures, including both diagnostic coronary angiography and PCI, with further details of early RAO, occurring < 24 h post-procedure or during the hospital stay, and late RAO, occurring > 24 h post-procedure. Subgroup analysis was executed to evaluate the effects of the following predictors on the primary endpoint: (1) studies performed in the USA vs. elsewhere, (2) size of catheter during coronary procedure <6 Fr vs. 6 Fr vs. >6 Fr, (3) Doppler ultrasound vs. non-Doppler ultrasound recognition of RAO, (4) PCI vs. coronary angiography (CAG), (5) high vs. low dose of low-molecular-weight heparin (LMWH), (6) LMWH vs. novel oral anticoagulants (NOACs), and (7) trans-radial hemostatic band compression duration.

### 2.6. Statistical Analysis

A meta-analysis was conducted to obtain a pooled estimate of the RAO rate across all cohort studies. In contrast, each individual study reported the rate of RAO in percentages. Statistical heterogeneity was assessed using I2 statistics, and due to significant heterogeneity, a random-effect meta-analysis was performed using the Mantel–Haenszel method to obtain a pooled estimate. The Cochran Q test was used to measure heterogeneity among subgroups, and a stratified analysis was performed to estimate the pooled rate of RAO between subgroups. Meta-regression analysis assessed the relationship between early and late RAO and age, catheter size, PCI, and angiography. Publication bias was evaluated graphically using contour-enhanced funnel plots and the trim-and-fill method. To further evaluate publication bias at a very sensitive level, the Doi plot and Luis Furuya-Kanamori asymmetry index were used. Sensitivity analysis was also conducted using a leave-one-out meta-analysis to examine the impact of each study on the overall effect-size estimate and to identify relevant studies.

## 3. Results

**Figure 2:**
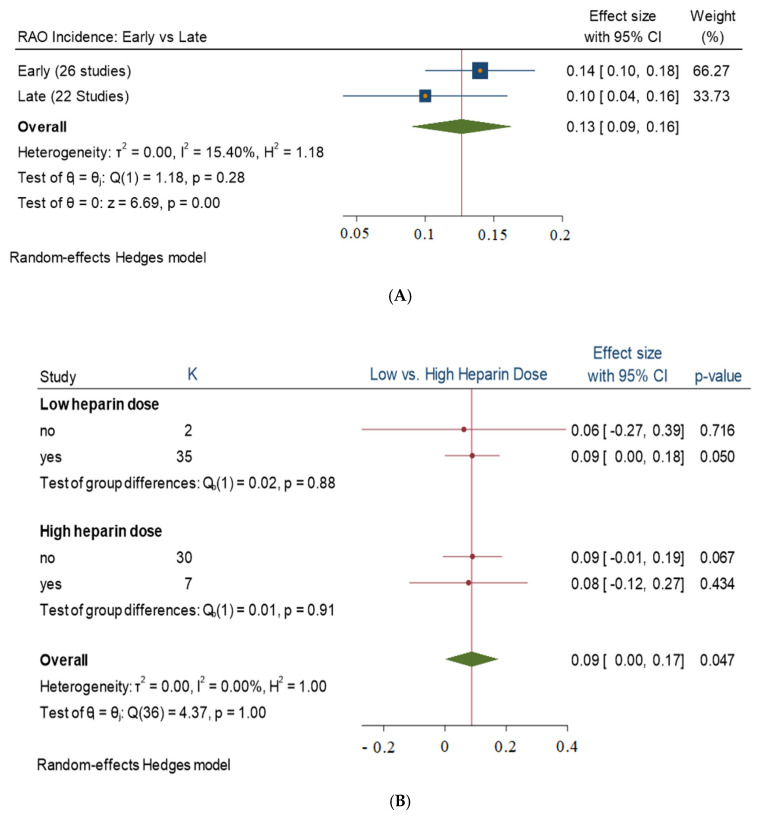
(**A**) Early and late RAO incidence. (**B**) Low-ose heparin vs. high-dose heparin. Heterogeneity assessment: I^2^ = 61.37%, *τ*^2^ = 0.85, H^2^ = 2.59, *p* < 0.001.

This meta-analysis included 41 studies with a total of 30,020 patients whose mean age was 61.80 ± 22.64 years. The overall incidence rate of RAO was 13%, with a 95% confidence interval (CI) of 0.09–0.16. The incidence of early RAO was 14% in 26 studies, with a 95% CI between 0.10 and 0.18. The incidence of late RAO was 10% in 22 studies, with a 95% CI of 0.04–0.16 (Figure 2A). The evaluation of RAO incidence occurred from 24 h to to 12 months after the procedure. The catheter size used in the studies ranged from 4 Fr to 8 Fr, with 2 studies using 4 Fr, 23 studies using 5 Fr, 35 studies using 6 Fr, 9 studies using 7 Fr, 1 study using 7.5 Fr, and 3 studies using 8 Fr.

### 3.1. Stratified Analysis

We conducted a stratified analysis to compare the use of Doppler ultrasound and non-Doppler ultrasound methods (clinical palpation, reverse Barbeau test, radial artery arteriogram) in assessing the incidence of RAO, and found that the pooled incidence of RAO was higher in the 31 studies that used Doppler US for RAO assessment (10%, 95% CI 0.00, 0.17) than in those that used non-Doppler ultrasound methods (5%, 95% CI −0.12, 0.22) (Supplemental Figure S1). Additionally, the average incidence rates of early and late RAO were compared for catheter sizes of <6, 6, and >6 Fr. It was observed that the incidence of RAO decreased with an increase in catheter size. The average incidence rates of early RAO in studies with catheter sizes of <6 Fr, 6 Fr, and >6 Fr were 9.8%, 9.4%, and 8.8%, respectively. The average incidence rates of late RAO in studies with catheter sizes of <6 Fr, 6 Fr, and >6 Fr were 8.4%, 7.8%, and 1%, respectively.

Moreover, a descriptive analysis of the included studies revealed a higher median incidence of early RAO in US studies (10%) compared to non-US studies (5.5%) and a lower median incidence of late RAO in US studies (<0.01%) than in non-US studies (2%). The forest plot illustrates the overall RAO incidence by study location, which showed no significant heterogeneity among the studies, with heterogeneity estimates of I^2^, *τ*^2^, and H^2^ of 0.00%, 0.00, and 1.00, respectively. The incidence of RAO for non-US studies was higher than that of US studies, but the confidence intervals overlapped 0.10 with 95% CI of 0.00, 0.19 and 0.05 with 95% CI of 0.13, 0.24, respectively (Supplemental Figure S2). These findings provide valuable insights into the incidence of RAO in studies conducted using different methods and across various locations.

### 3.2. Subgroup Analysis

**Figure 3:**
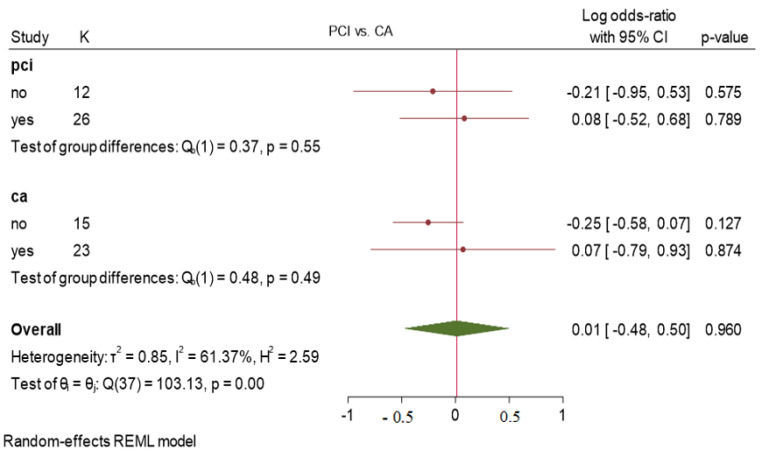
Subgroup analysis (PCI vs. CA). Heterogeneity assessment: I^2^ = 61.37%, *τ*^2^ = 0.85, H^2^ = 2.59, *p* < 0.001.

Results obtained from the subgroup analysis comparing the use of low-dose versus high-dose unfractionated heparin and PCI versus coronary angiography did not show any statistically significant difference in the incidence of RAO, with *p* values of 0.86, 0.63, 0.55, and 0.49, respectively. The overall incidence of RAO was 0.01 with a wide 95% confidence interval. Heterogeneity assessment showed high levels of variability among the studies (Figure 2B and Figure 3).

**Figure 4:**
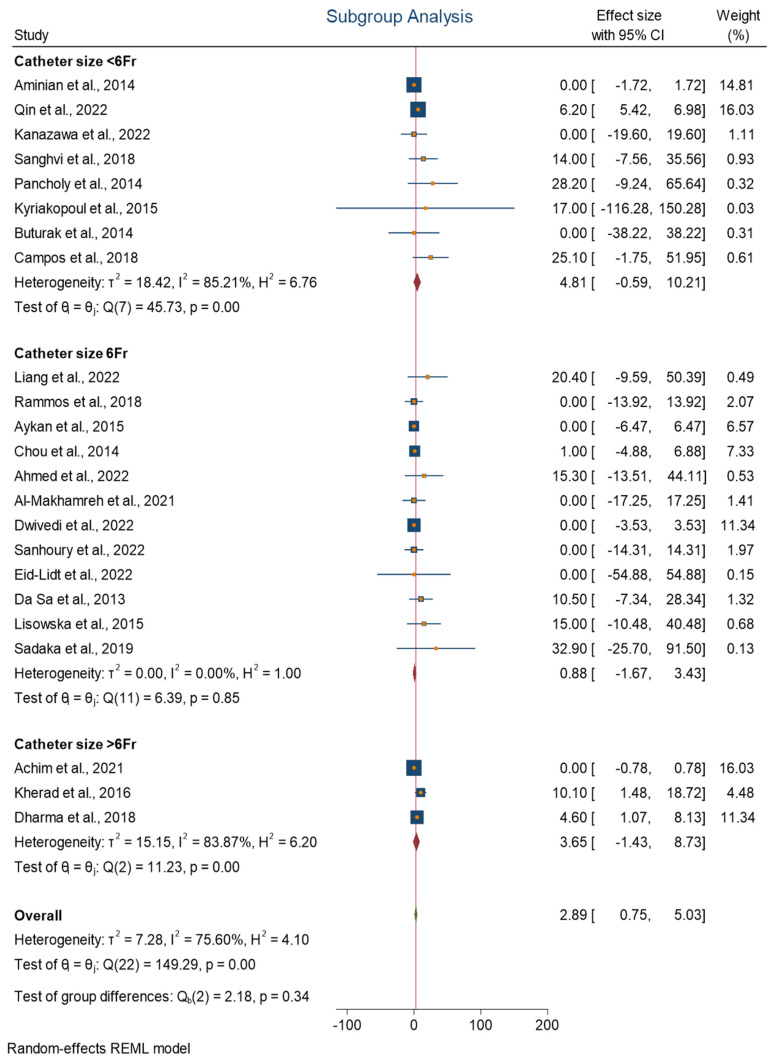
Forest plot of the association between different catheter sizes and the incidence of RAO.

The subgroup analysis depicted in Figure 4 presents a forest plot that examines the association between different catheter sizes and the incidence of RAO. The overall incidence of RAO was 2.89%, with a 95% CI of 0.75 to 5.03. The heterogeneity assessment yielded a value of H^2^ = 4.10, I^2^ = 75.60%, and *τ*^2^ = 7.28. The forest plot revealed varying incidence rates for three categories of catheter sizes, namely, <6 Fr, 6 Fr, and >6 Fr, with respective H^2^, I^2^, and *τ*^2^ values of 6.76, 85.21%, and 18.42; 1.00, 0.00%, and 0.00; and 6.20, 83.87%, and 15.15. However, differences among the groups were insignificant, with a *p*-value of 0.34. The forest plot further showed that the incidence of RAO decreased from 4.81 in catheter size < 6 Fr to 0.88 in catheter size of 6 Fr and then increased to 3.65 in catheter size > 6 Fr.

**Figure 5:**
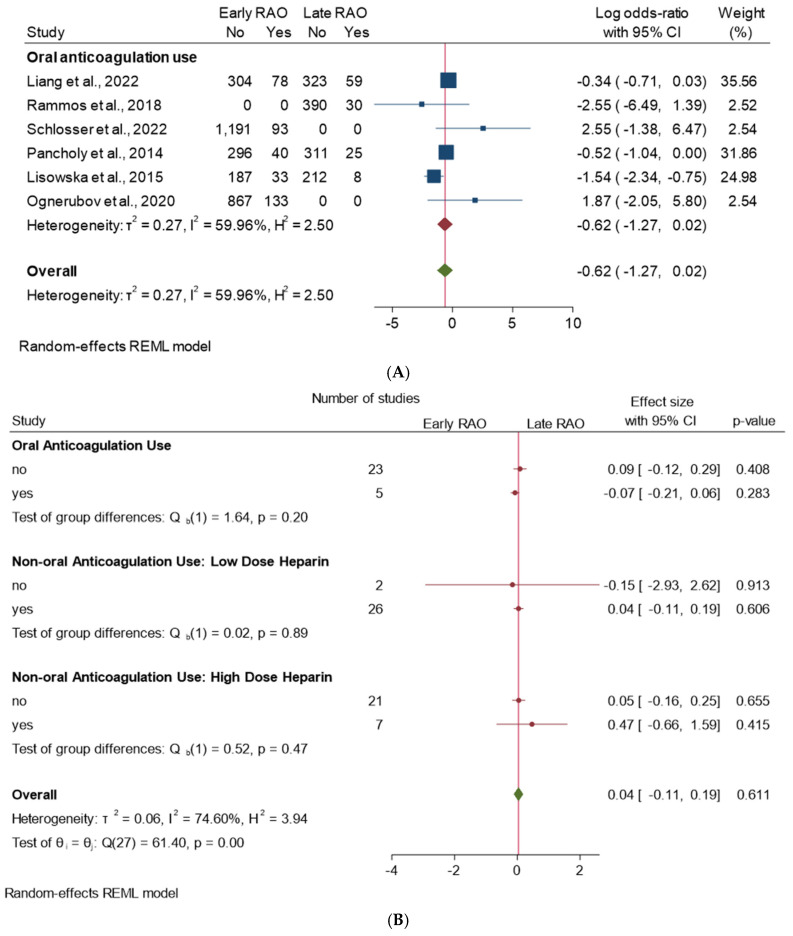
(**A**) (**panel superior**): Forest plot of the incidence of early and late RAO for interventional procedures using oral anticoagulation. (**B**) (**panel inferior**): Forest plot of the incidence of early and late RAO for interventional procedures using oral anticoagulation versus LMWH (low vs. high).

The forest plot in Figure 5A shows the results of a meta-analysis that examined the use of oral anticoagulation on the incidence of early and late RAO across six studies. The plot indicates that there is moderate heterogeneity between the included studies, as indicated by the I^2^ value of 59.96%. This suggests that the results of the individual studies are not entirely consistent with each other. The overall estimated effect size, represented by the diamond at the bottom of the plot, is −0.62 (62%), indicating that the use of oral anticoagulation is associated with a decrease in the incidence of RAO. The negative value indicates a risk reduction. However, it is important to note that the 95% CI for the effect size is relatively wide (−1.25–0.02) and includes negative values, which suggests that the difference may not be statistically significant.

**Figure 6:**
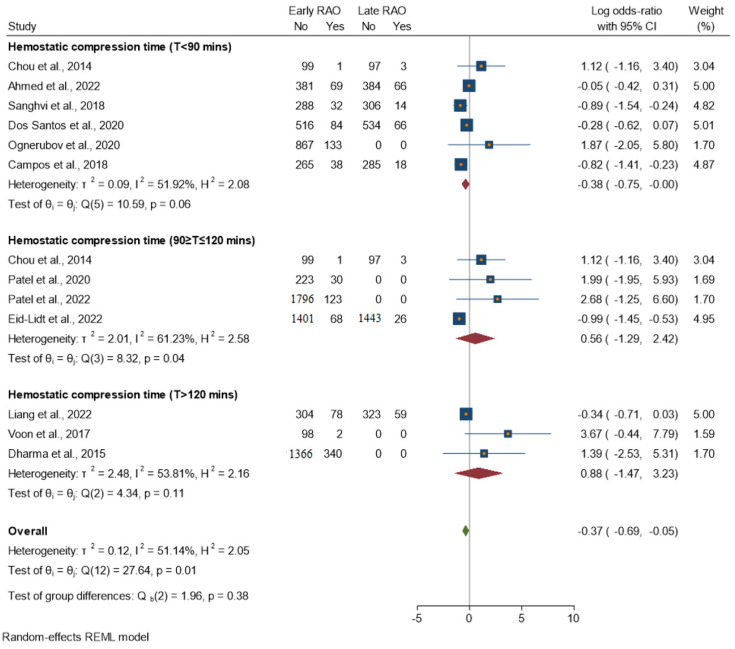
Forest plot of studies assessing the impact of hemostatic compression times on the incidence of early versus late RAO, summary log odds ratio with 95% confidence interval (CI), and weight (%) of each study.

The plot in Figure 5B indicates that there is some heterogeneity between the studies that compared oral anticoagulation to LMWH, as indicated by the I^2^ value of 74.6%. However, this heterogeneity is not statistically significant, as indicated by the *p*-value of 0.611. The overall estimated effect size, represented by the diamond at the bottom of the plot, is 0.04 (4%), suggesting a small difference in RAO incidence between the two treatment groups. However, it is important to note that the confidence interval for the effect size is relatively wide (−0.11–0.19) and includes the null value of 0, suggesting that the difference may not be statistically significant. Figure 6 shows an analysis of studies assessing the impact of hemostatic compression duration on early versus late RAO incidence. Overall, moderate heterogeneity is seen, with an I^2^ value of 51.14%. The test of no differences among the groups was rejected, with a chi-squared test statistic (Q) of 27.64 and a *p*-value of 0.01.

- T < 90 min: Moderate heterogeneity (I^2^ = 51.92%). The homogeneity study-specific effect-size test is not rejected, with a chi-squared test statistic (Q) of 10.59 and a *p*-value of 0.06.
- T 90–120 min: Moderate heterogeneity (I^2^ = 61.23%). The test of homogeneity study-specific effect size is rejected, with a chi-squared test statistic (Q) of 8.32 and a *p*-value of 0.04.
- T > 120 min: Moderate heterogeneity (I^2^ = 53.81%). The homogeneity study-specific effect-size test is not rejected, with a chi-squared test statistic (Q) of 8.32 and a *p*-value of 0.11.

**Figure 7:**
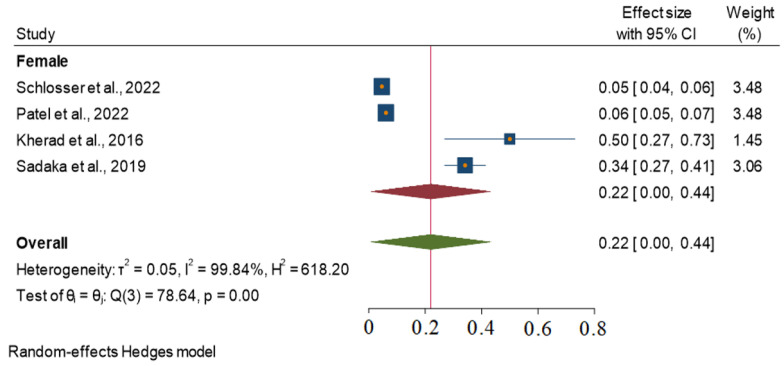
Female gender and RAO incidence across the studies.

A subgroup analysis of 4 out of the 41 studies included in the analysis identified female gender as a significant predictor of RAO incidence. The effect of female gender as a predictor of the overall incidence of RAO was found to be significantly heterogeneous across the four studies, with an I^2^ value of 99.84% and *p*-value < 0.001. The overall effect size of the female gender as a predictor was 0.22 with a 95% CI of 0.00–0.44. These findings suggest that there is significant variation in the effect of female gender on RAO incidence across the studies (Figure 7).

### 3.3. Heterogeneity Assessment

To assess heterogeneity among early and late RAO effect sizes, we utilized the Galbraith plot. Supplemental Figure S3a displays the standardized standard effect size versus precision of the 26 studies (represented by blue dots) analyzed for early RAO incidence, with the green line representing no effect. None of the studies fell below the green line and were far from the *y*-axis, indicating high precision. The red line indicates the overall effect size (0.14), and the shaded gray region represents the 95% CI. In sum, 2 of the 26 studies lay outside the 95% CI. In Supplemental Figure S3b, the standardized standard effect size versus precision of the 22 studies (represented by blue dots) analyzed for late RAO incidence is displayed, with no studies falling below the green line. Like Supplemental Figure S3a, the studies were far from the *y*-axis, indicating high precision. The red line indicates the overall effect size (0.10), and the shaded gray region represents the 95% CI. Just 1 of the 22 studies lay outside the 95% CI. Overall, the Galbraith plot indicates that there was no significant heterogeneity among studies assessing early or late RAO incidence.

### 3.4. Meta-Regression

Out of the 41 studies reviewed, 34 provided information on predictors of RAO, which is summarized in Supplemental Table S2. The results of a meta-regression analysis presented in Supplemental Table S5 showed that age is a strong predictor of RAO, particularly for early RAO (*p* = 0.006). This was supported by a bubble plot (Supplemental Figure S4), which is a scatterplot of the observed effect sizes against the moderator (overall mean age), along with the predicted regression and confidence interval lines. The plot also includes the predicted 95% confidence intervals. The plot indicates that the log odds ratio for RAO increases with age and identifies a couple of outlying studies, but their influence was small compared to other studies. However, only one study with a large bubble above the regression line fell completely outside the predicted 95% CI. Overall, these findings suggest that age is a strong predictor of RAO, particularly early RAO.

### 3.5. Publication Bias

The analysis for small study effects was performed using a contour-enhanced funnel plot, which showed no evidence of such an effect. Blue dots represented the studies included in the analysis. Additionally, the Egger test, which is a regression-based method for assessing small-study effects in a random-effect model, was not statistically significant (*p* = 0.599 and z statistic of 0.53) for any included study (Supplementary Figure S5). The Doi plot and Luis Furuya-Kanamori asymmetry index (LFK index) were used to assess publication bias. Of the 41 studies, 38 were included in the analysis, while 3 were excluded. An LFK index of 0.38 with a symmetrical plot was obtained, indicating no evidence of publication bias (Supplementary Figure S6).

## 4. Discussion

The present study conducted a systematic review and meta-analysis of studies investigating the incidence of RAO following invasive coronary procedures. Subgroup analyses were conducted to investigate the effect of different factors on the incidence of RAO. The findings from this analysis suggest that using Doppler ultrasound for RAO assessment is associated with a higher pooled incidence of RAO compared to non-Doppler ultrasound methods. This finding is consistent with a previous study that found that the use of Doppler ultrasound increased RAO detection significantly compared to non-Doppler ultrasound methods [9].

The meta-analysis found that compression time of <90 min was associated with a reduced incidence of RAO. However, the analysis also found moderate heterogeneity (I^2^ = 51.92%), which suggests that there was some variation in the effect sizes across the studies included in the analysis. At the same time, TR band compression time > 120 min, i.e., 2 h, was also associated with reduced RAO, but there was moderate heterogeneity. As indicated by the chi-squared test statistic (Q) of 8.32 and a *p*-value of 0.11, the homogeneity test suggests that the heterogeneity observed among the studies is not statistically significant. Maqsood et al., in their current study, mentioned that a duration of 2 h for achieving hemostasis provides an optimal balance between efficacy in preventing radial artery occlusion and safety in preventing access site complications such as hematoma and rebleeding [10].

The analysis also found that the incidence of early and late RAO decreased as catheter size increased. In particular, using a catheter size of 6 Fr may significantly decrease the incidence of RAO. This result is not consistent with previous studies that have reported a higher incidence of RAO with larger catheters [11,12,13]. Using larger catheters may reduce the incidence of RAO by decreasing the pressure on the arterial wall and reducing the risk of arterial spasms. The analysis also revealed differences in the incidence of RAO between studies conducted in the US and those conducted in other countries. The median incidence of early RAO was higher in studies conducted outside of the US compared to studies conducted within the US. This finding is consistent with a previous study that found a higher incidence of RAO in studies conducted outside of the US [14,15]. The reasons for these differences are not clear, but may be related to differences in patient populations and other factors and can limit the generalizability of the findings [15].

The results in this study suggest that the use of oral anticoagulation during interventional procedures may reduce the incidence of RAO. This has also been suggested in a recent best-practice review article [16], which also found that patients on chronic anticoagulation with a higher dose of LMWH showed reduced RAO. Still, our study could not confirm this, because most patients in our study were commenced on oral anticoagulation after the procedure. However, when compared with LMWH, there was significant heterogeneity in the incidence of RAO. This may be due to differences in study design, patient characteristics, and procedural factors that should have been accounted for in this analysis.

The subgroup analysis examined the impact of different doses of LMWH and different imaging techniques on the incidence of RAO after the vascular intervention. The results revealed no statistically significant differences based on low-dose versus high-dose heparin or PCI versus coronary angiography. This has been proven by two other studies on dosage in PCI and coronary angiography, respectively [17].

In diagnostic CAG, we use less heparin (5000 IU usually), less Fr (5 Fr in many places), much less procedural duration, and shorter pressure bandage time (always shorter when low doses of heparin are administered according to the manufacturer recommendations) whereas in PCI, at least 6 Fr (glide slender 7 Fr has an outer diameter of 6 Fr, which de facto is 6 Fr), more heparin (usually 100 IU/kg), longer procedural time and longer pressure bandage compression time, and still this study showed no statistical difference. A study by Hahalis et al. suggested that a higher dose of heparin than the standard dose in coronary angiograms significantly reduces the incidence of RAO [18]. But a trial done in ACS patients undergoing PCI with low dose vs. standard dose of unfractionated heparin showed no difference in vascular site complications [17].

The subgroup analysis of studies that used female gender as a predictor of RAO revealed significant heterogeneity in the effect of female gender on RAO incidence. This finding is consistent with previous studies that have reported mixed results regarding the association between female gender and RAO [19,20]. Potential factors influencing the occurrence of RAO in females are a smaller radial artery diameter, differences in vascular reactivity, hormonal influence, anatomical variations (such as tortuosity), etc. Further research is needed to determine the factors that underlie this heterogeneity.

The meta-regression analysis (Supplemental Table S4) showed that age is one of the strongest predictors of RAO, especially early RAO. A *p*-value of 0.006 means that developing RAO increases with age. This finding is consistent with previous studies that have shown a significant association between age and the incidence of RAO [13,19]. Health-care providers should be aware of this risk factor when performing invasive coronary procedures.

### 4.1. Potential Application of These Results

This meta-analysis provides critical insights into the incidence and predictors of RAO following angiographic procedures, highlighting early (14%) and late (10%) RAO rates. These findings can inform clinical guidelines by identifying female sex and age as significant predictors, aiding in the stratification of high-risk patients. The demonstrated efficacy of using Doppler imaging for RAO detection emphasizes the need for its routine implementation. The analysis reveals a substantial reduction in RAO incidence with the use of larger catheters (6 Fr), suggesting a reconsideration of catheter size in practice. Furthermore, while both oral anticoagulation and LMWH reduce RAO, the lack of statistical significance points to the need for further research to optimize anticoagulant protocols.

### 4.2. Limitations

Several limitations should be acknowledged. Firstly, there may be heterogeneity among the included studies in terms of patient populations, procedural techniques, and definitions of RAO, which could affect the generalizability of the results. Secondly, the reliance on Doppler imaging for RAO detection varies across studies, possibly leading to inconsistencies in reported incidence rates. Additionally, the analysis of non-US studies showing higher RAO incidence might reflect differences in health-care systems, operator experience, or procedural standards that are not fully accounted for. The lack of statistical significance in comparing oral anticoagulation with LMWH, despite observed trends, suggests that larger, well-designed randomized controlled trials are needed to draw definitive conclusions.

Patent hemostasis is a procedure that was introduced by the PROPHET study in 2008 [21], and it was found to be associated with a significantly lower incidence of RAO, either by performing the original procedure, guided by mean arterial pressure (MAP) [22], or with the implementation of a rapid deflation technique (RDT) [23]. Although patient hemostasis has been shown to significantly reduce the risk of RAO, we did not include this variable in our study. Additionally, other factors that have been suggested to influence the incidence of RAO, such as renal failure (which increases the risk) [24] and the use of statins (which may reduce the risk) [25], were not explored in this meta-analysis.

Finally, publication bias and the quality of the included studies could influence the overall findings, necessitating cautious interpretation and validation through further research.

## 5. Conclusions

This meta-analysis showed that early (within 24 h) and late (>24 h) incidence of RAO were 14% and 10%, respectively. Additionally, our findings suggest that female sex and age are possible predictors of RAO. Use of Doppler images increases detection of RAO, and incidence of RAO is high in non-US studies. A larger catheter, especially 6 Fr, was associated with a substantial reduction in the incidence of RAO. At the same time, the use of oral anticoagulation and the appropriate dosage of LMWH does reduce RAO, but comparison between them showed no statistical significance.

## Supplementary Materials

The following supporting information can be downloaded at: https://www.mdpi.com/article/10.3390/jcm13195882/s1, Figure S1: Use of Doppler and Non-Doppler methods in assessing the incidence of RAO; Figure S2: The incidence of RAO for non-US studies was higher than that of US studies; Figure S3: Galbraith Plot – to assess the Heterogeneity; Figure S4 Bubble Plot for publication Bias; Figure S5: Funnel Plot for graphical diagnostics of small-study effects; Figure S6: Doi Plot & LFK index of Publication Bias; Table S1: Study designs and characteristics: Table S2: RAO predictors and outcomes; Table S3: Risk-of-bias assessment for randomized trials (RoB 2); Table S4: NEWCASTLE - OTTAWA Quality Assessment for Observational Studies; Table S5: Meta-Regression Analysis (Early vs Late RAO). References [26,27,28,29,30,31,32,33,34,35,36,37,38,39,40,41,42,43,44,45,46,47,48,49,50,51,52,53,54,55,56,57,58,59,60,61,62,63] are cited in the supplementary materials.
