# Supplementary material for: Incidence and Predictors of Early and Late Radial Artery Occlusion after Percutaneous Coronary Intervention and Coronary Angiography: A Systematic Review and Meta-Analysis
Source: J Clin Med. 2024 Oct 2;13(19):5882. doi: 10.3390/jcm13195882 (PMC11477189; doi:10.3390/jcm13195882)
Supplement: Supplementary file 1 [file jcm-13-05882-s001.zip › jcm-3132793-supplementary.pdf]

Journal of
Clinical Medicine

MDPI

SUPPLEMENTARY MATERIAL

Incidence and predictors of Early and Late Radial Artery Oc-clusion After Percutaneous Coronary Intervention and Coronary Angiography: A Systematic Review and Meta-Analysis

Aisha Khalid [1], Hans Mautong [2], Kayode Ahmed [3], Zaina Aloul [4], Jose Montero-Cabezas MD PhD [5] and Silvana Marasco MD PhD

1 Harvard Medical School, MA, United States
2 Universidad Espíritu Santo, Samborondón, Ecuador
3 The University of Texas MD Anderson Cancer Centre, USA
4 Cardiff University School of Medicine, Wales, UK
5 Department of Cardiology. Leiden University Medical Center. The Netherlands
6 The Alfred Health, Melbourne, Australia

TABLE OF CONTENTS

Supplemental Figure S1. Use of Doppler and Non-Doppler methods in assessing the incidence of RAO

Supplemental Figure S2. The incidence of RAO for non-US studies was higher than that of US studies

Supplemental Figure S3. Galbraith Plot – to assess the Heterogeneity.

Supplemental Figure S4. Bubble Plot for publication Bias.

Supplemental Figure S5. Funnel Plot for graphical diagnostics of small-study effects. [Contour-enhanced funnel plot for publication bias at different CIs- 90%, 95%, and 99% (or **α** = 0.1, 0.05, and 0.01, respectively). This contour-enhanced funnel plot is a scatterplot depiction of study-specific effect sizes (log odds-ratio) on the horizontal axis against the measures of study precision (standard error) on the vertical axis. The symmetry of the plots shows the absence of small-study effects (rules out publication bias). [Blue dots in the plots represent studies included in the analysis]. The null hypothesis of no small-study effects or symmetry of the funnel plot is not rejected at the 5% significance level with a z statistic of 0.53 and a p-value of 0.599]

Supplemental Figure S6. Doi Plot & LFK index of Publication Bias. [The Doi plot and Luis Furuya-Kanamori asymmetry index (LFK index) were used to assess publication bias. 38 of the 41 studies were included, and 3 were excluded. An LFK index of 0.38 with a symmetrical plot was obtained and is consistent with the absence of publication bias. A Doi plot is more sensitive in detecting the presence of publication bias in comparison to an Egger test (7–9).

Supplemental Table S1. Study designs and characteristics

Supplemental Table S2. RAO predictors and outcomes.

Supplemental Table S3. Risk-of-bias assessment for randomized trials (RoB 2).

Supplemental Table S4. NEWCASTLE - OTTAWA Quality Assessment for Observational Studies .

Supplemental Table S5. Meta-Regression Analysis (Early vs Late RAO)

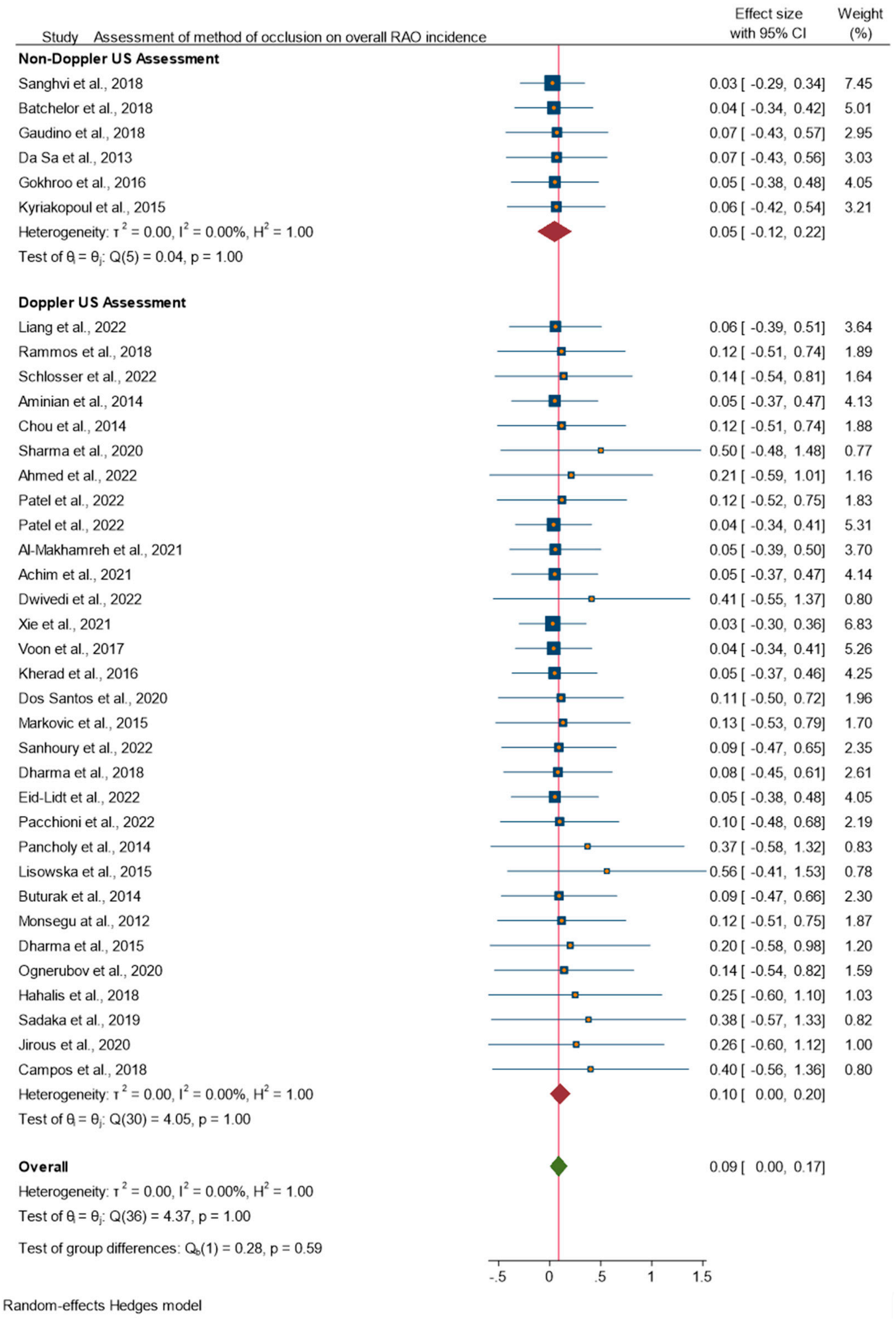

Supplemental Figure S1. Use of Doppler and Non-Doppler methods in assessing the incidence of RAO.

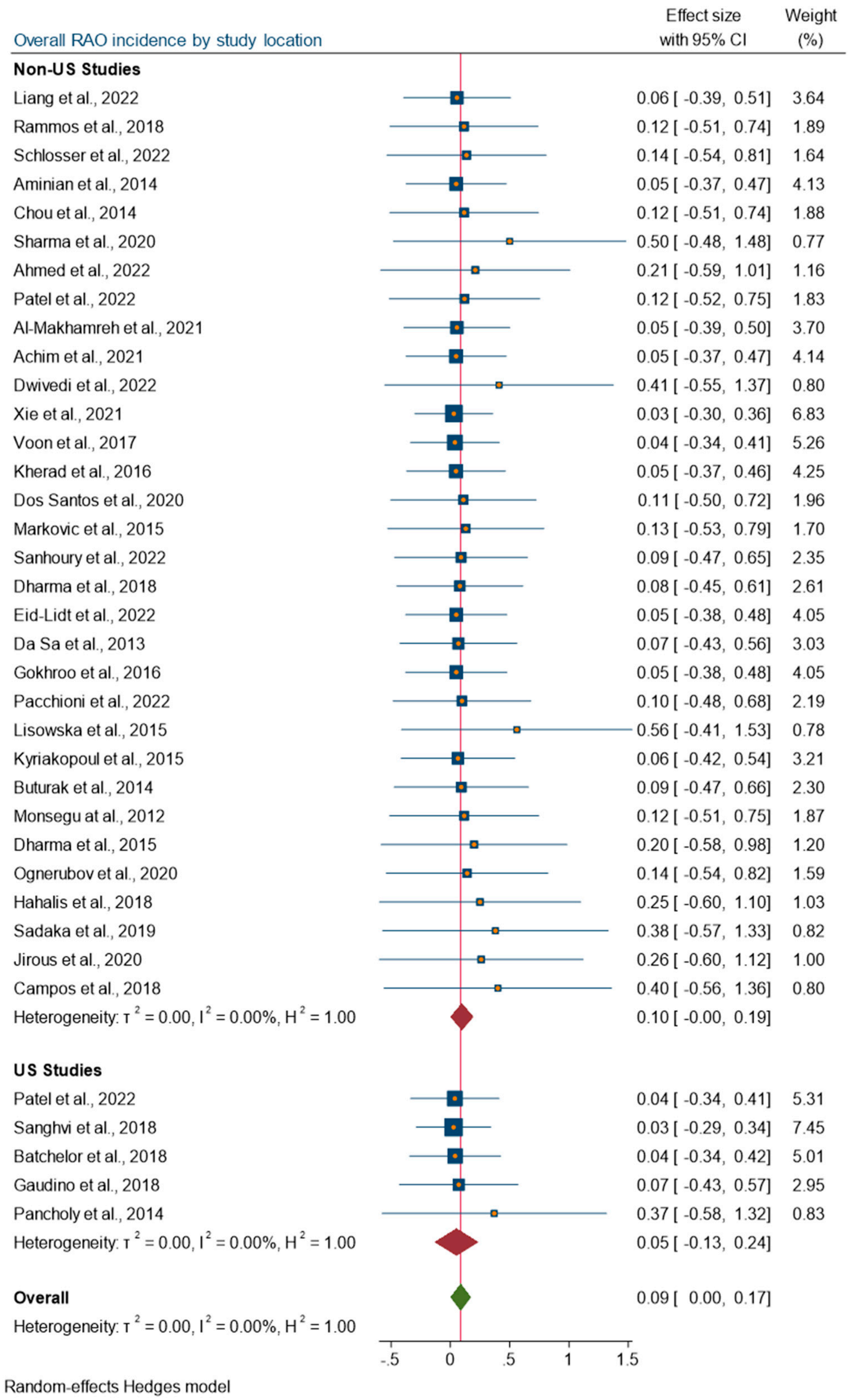

Supplemental Figure S2. The incidence of RAO for non-US studies was higher than that of US studies

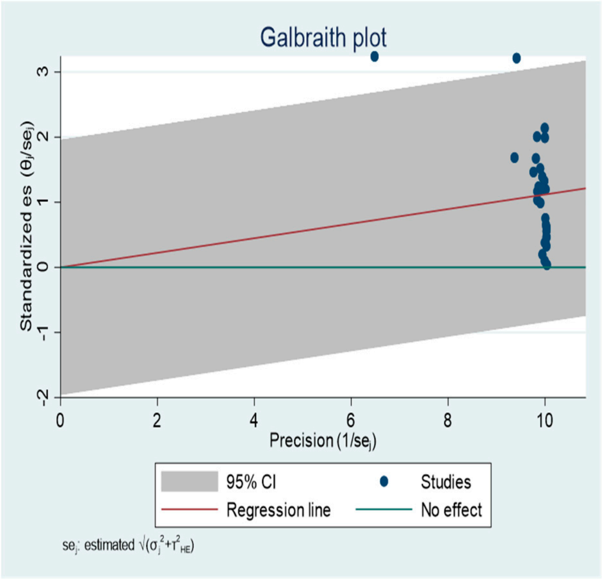

(a) **Early RAO**

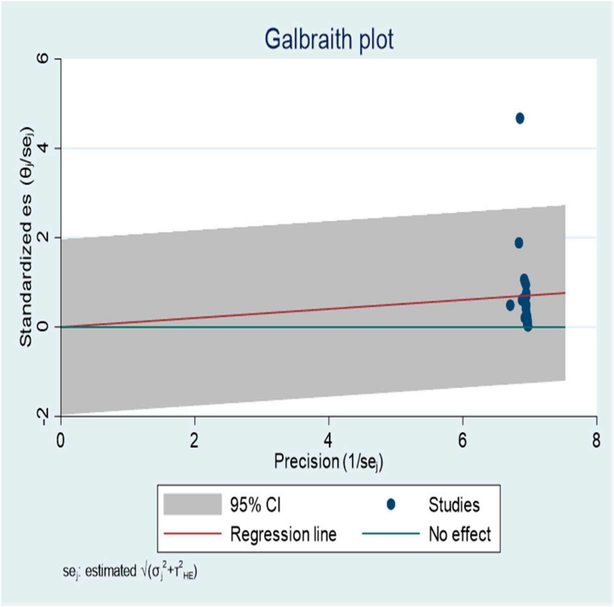

(b) **Late RAO**

Supplemental Figure S3. Galbraith Plot – to assess the Heterogeneity.

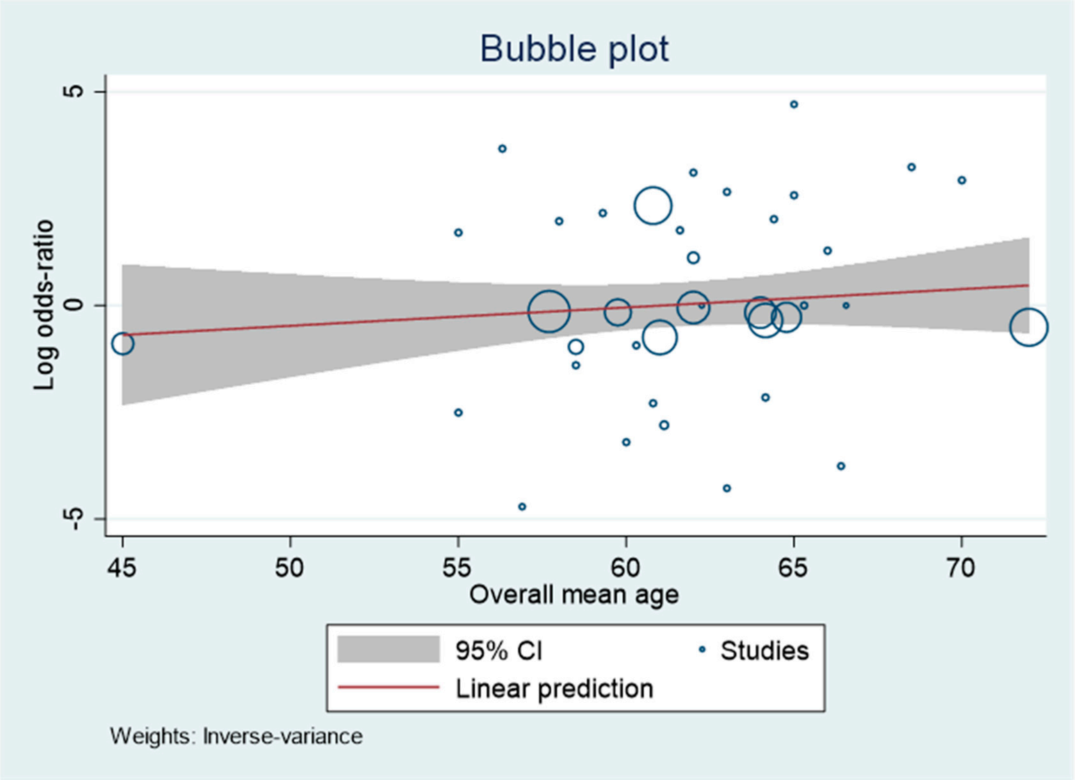

Supplemental Figure S4. Bubble Plot for publication Bias.

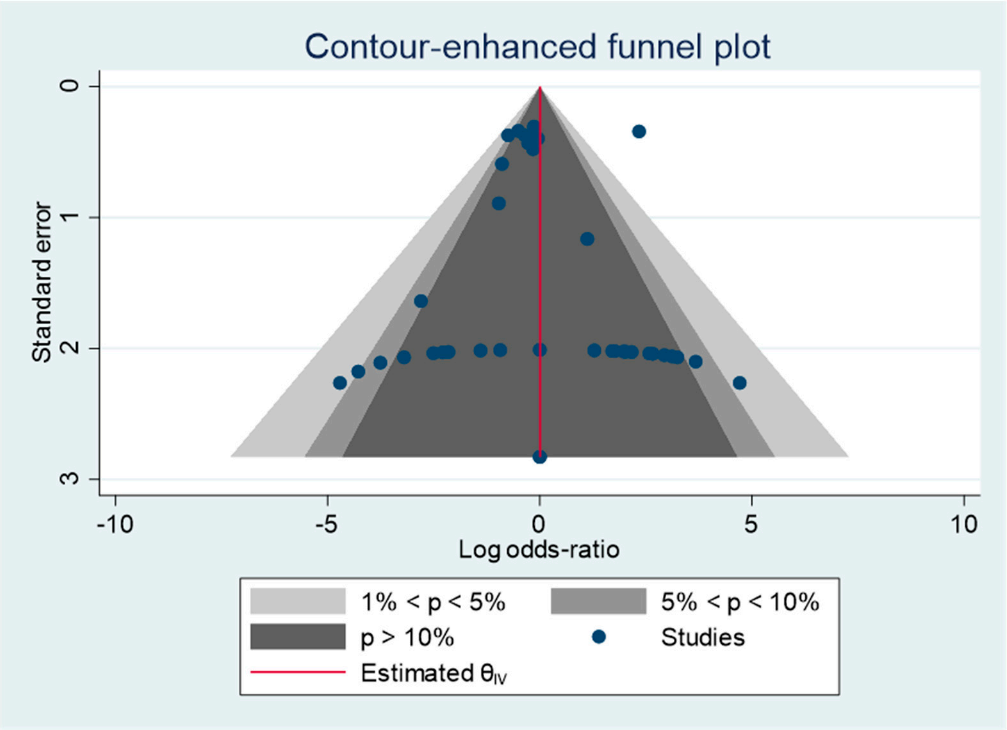

Supplemental Figure S5. Funnel Plot for graphical diagnostics of small-study effects.

[Contour-enhanced funnel plot for publication bias at different CIs- 90%, 95%, and 99% (or **α** = 0.1, 0.05, and 0.01, respectively). This contour-enhanced funnel plot is a scatterplot depiction of study-specific effect sizes (log odds-ratio) on the horizontal axis against the measures of study precision (standard error) on the vertical axis. The symmetry of the plots shows the absence of small-study effects (rules out publication bias). [Blue dots in the plots represent studies included in the analysis]. The null hypothesis of no small-study effects or symmetry of the funnel plot is not rejected at the 5% significance level with a z statistic of 0.53 and a p-value of 0.599]

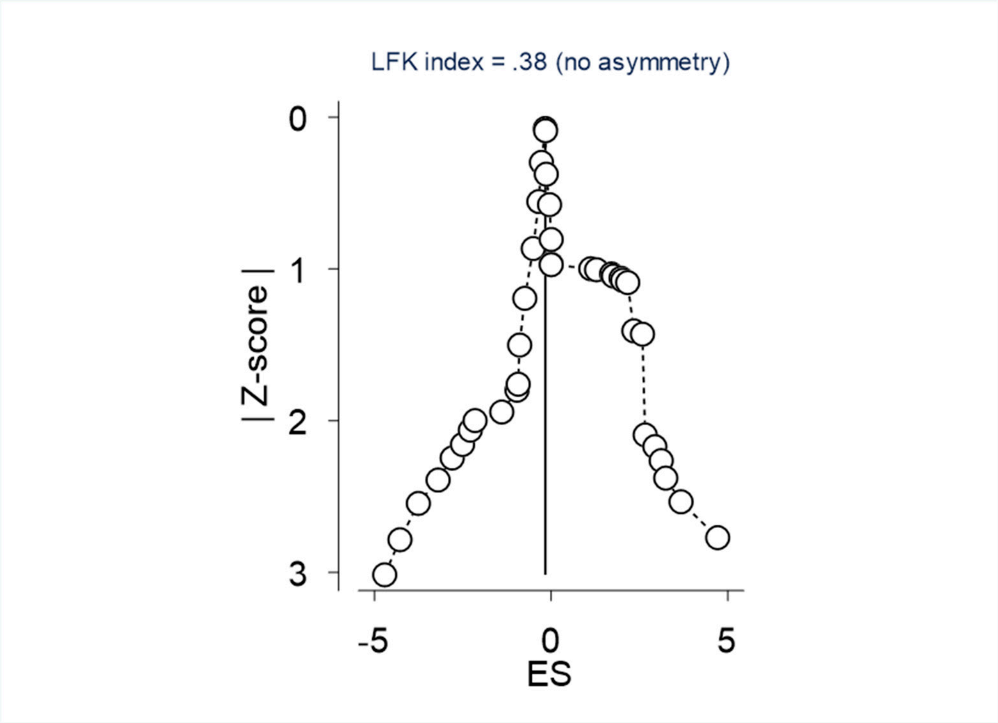

Supplemental Figure S6. Doi Plot & LFK index of Publication Bias. [The Doi plot and Luis Furuya-Kanamori asymmetry index (LFK index) were used to assess publication bias. 38 of the 41 studies were included, and 3 were excluded. An LFK index of 0.38 with a symmetrical plot was obtained and is consistent with the absence of publication bias. A Doi plot is more sensitive in detecting the presence of publication bias in comparison to an Egger test (7–9).

Supplemental Table S1. Study designs and characteristics

Author, Year	Study Type/Country	Sample size	RAO within 24 hours	RAO within a month	RAO by catheter size (Fr)	RAO follow-up time	Assessment of Occlusion	Others
Liang et al., 2022 [29]	RCT/China	382	No significant difference in the incidence of 24-hour RAO (8.9% versus 11.5%; *P*=0.398)	1-month RAO (3.8% versus 11.5%; *P*=0.011) was significantly reduced; (odds ratio, 0.22 [95% CI, 0.08-0.65]; *P*=0.006)	6 Fr	4 weeks	Doppler US	Hemostatic compression
Roy et al., 2021 [30]	Case series/USA	4			6 Fr	5 weeks	Arterial Doppler US	Oral anticoagulation instead of LMWH
Rammos et al., 2018 [31]	Single center case control study/ Germany	420	NA	30/420=7.1%	6 Fr	12 weeks	Duplex US	Control= no anticoagulation<br><br>treatment= LMWH / NOAC<br><br>RAO can be treated with anticoagulation
Schlosser et al., 2022 [19]	Prospective study/Germany	2004	4.6% Incidence rate (93/2004)	NA	5 Fr- 679<br><br>6 Fr- 1198<br><br>7 Fr-	30 days	Duplex ultrasonography	Strongest predictors: female and smoking 32% reperfusion after oral

					127			anticoagulation
Aykan et al., 2015 [32]	RCT/Turkey	459	N/A	Incidence of RAO in both groups: 15/459. RAO with 2500 IU heparin: 12/217. RAO with 5000 IU heparin: 3/242	6 Fr	4 weeks	Doppler USS	
Aminian, et al., 2014 [15]	Retrospective cohort/Belgium	113	N/A	RAO 1/113 - 0.88%	4=4 5=72 6=38	4 weeks	Doppler US	Patent hemostasis =101 Occlusive radial pressure=2
Chou et al.,2014 [33]	Observational study/China	100	incidence: 1/100. short compression: 0/50 (0%). conventional compression: 5/50 (10%).	incidence: 3/100. short compression: 0/50 (0%). conventional compression: 3/50 (6%)	6 Fr	8 weeks	Coloured Doppler US	Compared the compression techniques
Sharma et al., 2020 [34]	Open-label RCT/India	970	Early RAO:2% (10) group A and 13% (63) patients in group B (p- value <0.0001)	NA	5 Fr	1 day	Doppler US	Group A: dRA Group b: Conventional RA approach
Ahmed et al., 2022 [35]	Randomized duplex follow-up study (RCT)/Egypt	450	RAO 1-h post TR-band removal was significantly lower among patients of	At 1-month (0.7%, 8%, and 6%, respectively -p = 0.03)	6 Fr	1 month	Duplex ultrasound	Group A-TR band Group b-conventional hemostasis Group c-Simultaneous ulnar

			group C as compared to those of group A and B (1.3%, 6.7%, and 7.3%, respectively -p = 0.03).					and radial artery compression (SURC)
Patel et al., 2020 [36]	RCT/India	253	Early RAO: Double balloon band compression vs single balloon 1.6% vs 10.2%, respectively; P<.001	NA	5 Fr	1 day	Doppler US	Group 1 : Single balloon Band compression Group 2 double balloon band compression
Patel et al., 2022 [37]	Retrospective study/USA	2019	Early RAO in the one-bladder band group, with a 6-Fr slender introducer sheath, was 4.2%.<br><br>In the Two-bladder band, incidence is 1% of patients receiving a 6-Fr slender introducer sheath versus 0.9% in those receiving a 7-Fr slender introducer sheath (p = 0.68	NA	6- 72<br><br>7-427	1 day (24 hours)	Doppler Ultrasound	Mean Age= 63

Qin et al., 2022 [38]	Prospective observational cohort study/China	2316	The rate of RAO at 24 h after TRCA was (6.22%), which was evenly distributed among the single/dual-antiplatelet groups (6.2 vs. 6.2%, p = 0.963)	59.72% demonstrated self-recanalization & delayed occlusion occurred in 8/2172 patients (0.37%), which was not significantly different between the two groups (0.5 vs. 0.2%, p = 0.226)	5 Fr	30 days	2D & Doppler Ultrasound	
Kanazawa et al., 2022 [39]	Retrospective study/Japan	558	NA	RAO incidence in 55 patients (10%)	5 Fr	12 months	Radial artery angiograms	All patients were post PCI and received DAPT<br><br>Increase incidence in patient with lower BSA
Al-Makhamreh et al., 2021 [40]	Retrospective case-control study/Jordan	148	NA	13/148: 8.8% Late RAO	6 Fr	56 weeks	Coloured Doppler US	
Achim et al., 2021 [41]	Prospective observational (registry) study/ Hungary	1240	NA	RAO incidence: 5/1240 (only 0.4% of the entire study population).	5:852<br>6:269<br>7:11<br>8.5:4	12 months	Doppler Ultrasound	
Dwivedi et al., 2022 [11]	Single-center	1754	11.97%	NA/ND	5:378	NA/ND	Color Doppler Ultrasound	

	prospective study/India				6:136 7<br><br>7:9			
Xie et al., 2021 [42]	Retrospective study/ China	1063	NA	RAO at the access site (13 cases, 1.4%), forearm radial artery occlusion (4 cases, 0.4%),	5-381<br><br>6-682	7 days	Doppler	
Voon et al., 2017 [43]	RCT/Germany	100	incidence of RAO at 24 h (2% *vs* 0%) in both Safeguard Radial *vs* TR band groups)	6 week (0%, in both Safeguard Radial *vs* TR band groups)	6 Fr	6 weeks	Handheld Doppler Ultrasound	
Kherad et al., 2016 [44]	Safety/efficacy/Germany	18	50% (9/18) of the patients	NA	7.5 Fr	1 day	Color Doppler Ultrasonography	
Sanghvi et al., 2018 [45]	RCT/USA	320	Early RAO incidence (3.8% TRG vs. 6.28% SGG, p = 0.05)	Late RAO incidence : (1.9% TRG vs. 2.5% SGG; p = 0.21)	5 Fr:856<br>6 Fr:229	4 weeks	Reverse Barbeau's test	Comparison of two hemostatic compression devices (TRG vs SSG)
Santos et al., 2020 [46]	RCT/Brazil	600	Early RAO occurred in 24 (8%) in the TR Band group and 19 (6%) in the pressure-dressing group.	Late RAO: 5 patients (5%) in the TR Band group and 7 (6%) in the pressure-dressing group	5 Fr:318<br>6 Fr:282	4 weeks	Doppler USS	TR Vs Pressure dressing group
Markovic et al., 2015 [47]	Prospective Cohort/Germany	369	Incidence of early RAO 3.8%	NA	4:9<br>5:124<br>6:233	24 hours	Doppler US	

			(14/369) with no difference to 5 or 6 Fr (2.2 vs 4.0% p=0.56)		7:3			
Sanhoury et al., 2022 [48]	Observational study/Egypt	100	RAO-no statistical difference in dTRA (group 1) is 2/50 (4%) group 2 (TRA) 7/50 (14%) p=0.160	NA	6 Fr	24 hours	Doppler	dTRA vs Conventional TRA
Batchelor et al., 2018 [49]	RCT/USA	41	NA	Late RAO is infrequent and not different in 7fr= 2 vs 6Fr=1 (P=0.10)	6- 22<br>7- 19	90 days	Ultrahigh-resolution vascular ultrasound	Comparison of 6Fr vs 7Fr. All underwent TRA-PCI
Dharma et al., 2018 [50]	Post hoc analysis of a randomized trial/Indonesia	1706	Early RAO occurred in 16.0% of group A(Nitro) and 5.4% of group B(Verapamil) statistically non significant odds ratio [OR], 1.24; 95% confidence interval [CI], 0.51-3.02; P=.62)	NA	**Nitro group**<br>5-438<br>6- 250<br><br>**Vera pa Group**<br>5-58<br>6- 957<br>7- 2<br>8- 2	1 day	Doppler Ultrasound	Nitroglycerine Vs verapamil<br><br>Strongest predictor = Radial artery compression > 4 hours
Eid-Lidt et al., 2022 [51]	RCT/Mexico	1469	Total early RAO	Late RAO incidence:1.8 %	6 Fr	4 weeks	Plethysmography oximetry	3 groups A= standard

			incidence = 4.6% Individually A=3.6% B=5.5% C=4.7%<br><br>With no statistical significance group wise P = 0.387	Individually A=1.4% B=1.8% C=2.2% p=0.185			and confirmed by Doppler	patent hemostasis . B: ULTRA C= facilitated hemostasis
Gaudino et al., 2018 [52]	RCT/ USA	1036	NA	Radial artery graft occlusion 28/345 (8.1%) versus saphenous-vein group;61/307(19.9%) (hazard ratio, 0.44; 95% CI, 0.28 to 0.70; P<0.001)	6 Fr	5 Years	Angiography	Graft occlusion post CABG RA vs Saphenous vein
De Sa et al., 2013 [53]	Observational study/Brazil	228	Incidence of early RAO: 24/228(10.5 %) New introducers: 10/100 (10%) Vs Reprocessed introducers: 14/128 (10.9%)	Incidence of late RAO: 17/186(9.1%) New introducer 6/80 (7.5%) Vs Reprocessed introducers: 11/106 (10.4%)	6 Fr	4 weeks	Reverse Barbeue test	
Gokhroo et al., 2016 [54]	Retrospective cohort /India	1270	Incidence rate: 83/1270, 6.6% , RR:0.93, 95% CI	NA	5-6Fr	1 week	Clinically impalpable pulse at 1 week	1 wk follow-up, ulnar (6.1%) and radial routes (6.6%)- no

			1.26 , P value:0.65					sig difference. Absolute difference of 1.8% in incidence of spasm (6.9% in UA vs 8.7% in RA; *P*=.09).
Pacchioni et al., 2022 [55]	Retrospective Study/Italy	1163	Early RAO incidence:(0 of 213, 0% vs. 7 of 213, 3.3%, p = 0.0015)	NA	5=182 6=963 7 = 17	4 weeks	Doppler US	Distal vs Conventional artery assessment
Pancholy et al., 2014 [56]	Retrospective study/USA	336	Early RAO :warfarin group: 16/86 (18.6%). Early RAO in the heparin group: 24/250 (9.6%) p=0.024	Late RAO in the warfarin group: 12/86 (13.9%). Late RAO in heparin group: 13/250 (5.2%) p=0.01	5 Fr	4 weeks	Doppler US	
Lisowska et al., 2015 [24]	Prospective cohort/Poland	220	Early RAO: incidence 33/220 (15%).	Late RAO: Incidence 8/220 (13%)	6 Fr	52 weeks	2D-Colour Doppler US	
Kyriakopoulos et al., 2021 [57]	RCT/Greece	299	RAO rates at 24h: 12% in patients with conventional hemostasis vs. 5% with patent hemostasis	RAO rates at 30 days did not differ between the two groups. The rates of patent hemostasis in this study are similar to those in previous	5:150<br>6:149	30 days	pulse oximeter *vs* artery palpation	Control Vs Pulse OXy group

				studies (68.1% to 82.2%)				
Buturak et al.,2014 [58]	Prospective Study/Turkey	409	N/A	Long term RAO incidence: 67/243 (19.5%)	5 Fr: 183<br>6 Fr: 226	65 weeks	Doppler ultrasound	
Monsegu et al, 2012 [59]	Prospective cohort/Europe (8 centers)	574	Early RAO incidence :2 2/574 (3.8%)	NA	5 -363<br>6- 211	1 day	Color Doppler	
Dharma et al., 2015 [60]	RCT/ Germany	1706	Early RAO Incidence : 170/1706 (9.9%) nitro-glycerine arm: 70/853 (8.2%) placebo: 100/853 (11.7%)	NA	6 Fr- 1207<br>5 FR: 496<br>7 Fr: 2<br>8 Fr:1	1 day	Color Doppler	Intra-arterial administration of nitro-glycerine (500 µg) vs placebo post procedure<br>TR-band for hemostasis
Ognerubov et al., 2020 [61]	RCT/Russia	1000	Early RAO= **Prolonged group** 10.1% **Short group**- 3.2%	NA	6 Fr	1 day	Pulse oxy followed by doppler	Prolonged hemostasis Vs short hemostasis
Hahalis et al, 2018 [18]	RCT/Greece	1836	Early RAO: **High heparin dosage:** 3.2%(27)<br>**Low heparin Dosage:** 8.1%(75)		5- 1259<br>6-577	10 days	Doppler US	Low dose Heaprin Vs High dose Heparin
Sadaka et al, 2019 [20]	Prospective Study /Egypt	164	Early RAO: 54/164 (32/9%)	Late RAO 49/164 (29.9%)	6 Fr	24 Weeks	Doppler US	

Jirous et al, 2020 [62]	Prospective Cohort /Czech Republic	500	Early RAO: 2 (0.4%)	NAD	5 -370<br>6-130	1 Day	Doppler US Reverse Barbeau Test (RBT)
Campos et al, 2018 [63]	RCT, Brazil	303	Early RAO:<br>**G30:**<br>20/152<br>(13.2%)<br>**G60:**<br>18/151<br>11.9%	Late RAO<br>**G30:**<br>7/152<br>5.5%<br>**G60:**<br>11/151<br>8.2%	5 -190<br>6-113	30 days	Doppler US

Supplemental Table S2. RAO predictors and outcomes.

Author, Year	Predictors of RAO	Duration of the procedure	Age	Procedures	Use of anticoagulation	Outcome
Liang et al., 2022 [29]	**Early RAO:** **Age=**OR (0.97), CI (0.93-0.99), p=0.033 **Procedure duration=**OR (1.04), CI (1.01-1.08), p= 0.008 **RA diameter=** OR (0.40), CI (0.20-0.83), p= 0.014 **Late RAO:** **Age=**OR (0.95), CI (0.91-0.99), p=0.028 **RA diameter=**OR (0.29), CI (0.11-0.74), p= 0.009 **Procedure duration=**OR (1.06), CI (1.02-1.10), p= 0.007 **Heparin dosage=** OR (0.84), CI (0.73-0.96), p= 0.009	**Control=** 28.5 min **Rivaroxaban=**28.4	**Control**=64.0+/-10.0 **Rivaroxaban**=64.3+/-10.1	**PCI:**183 **CA:**199	All patients were pretreated with **aspirin and P2Y12 inhibitor**. An intra-arterial bolus of 200 µg **nitroglycerin** was routinely administered to prevent arterial spasm. **Unfractionated heparin of 2500 IU** was given for a diagnostic angiography, and in total 100 IU/kg was given for ad hoc intravascular ultrasounds or PCI.	Short-term postoperative anticoagulation with rivaroxaban did not reduce the rate of 24-hour RAO but improved 1-month RAO, because of higher recanalization of the radial artery.

Roy et al., 2021 [30]			65.25	**All have PCI**	Pre procedure standard UFH 5000 IU<br><br>Apixaban	Novel oral anticoagulants such as apixaban could offer significant benefit to patients with RAO
Rammos et al., 2018 [31]	**Non occlusive haemostasis:**<br>p>0/001	25.3 ± 15.45	55+/-10.48	Diagnostic mainly **(CA)**	5000 IE Heparin together with 0.2 ml nitroglycerin and 1.5 mg verapamil	study confirms the effectiveness of anticoagulation in therapy of RAO, while the addition of vasoactive medication does not seem to confer to additional benefit.
Schlosser et al., 2022 [19]	**Female:**<br>OR (2.91)<br>CI (1.90-4.45)<br>P:<0.001<br><br>**Smoking**<br>OR (2.39)<br>CI (1.51-3.76)<br>P:<0.001	27	70	**PCI:**788<br>**CA:**1216	Oral anticoagulation for 30 days to RAO and assess recanalization & Heparin as standard during procedure	The limited effectiveness of oral anticoagulation for treatment of radial artery occlusion suggests a primarily traumatic than thrombotic mechanism of this complication.

Aykan et al., 2015 [32]	**Female:** OR (66.13), CI (4.58-9.54), P:0.002 **Sheath removal time:** OR (1.49), CI (1.25-1.78), P:<0.001 **2500 IU Heparin:** OR (9.75), CI (1.19-79.9), P:0.034		**60**	All diagnostic angiograms CA- **459**	**One group** ;2500-IU heparin **Control:** 5000 IU Heparin 2 mg of diltiazem, 200 mcg nitroglycerin to **ALL PATIENTS**	The patients in the standard dose heparin group had lower RAO rates compared to the low dose group in this study.
Aminian, et al., 2014 [15]	Anatomical **variations** Small **Radial arterial diameter** (radial artery diameter in F=2.43-2.8 mm and in M=2.69-3.1mm)		63+/- 11	**PCI:** 35 **CA:**78	Heparin 5000 IU + nitroglycerine 200 microgram	Routine use of the Glidesheath Slender for TR coro- nary angiography and intervention is safe and feasible with a high rate of procedural success and a low rate of RAO.
Chou et al.,2014 [33]	Duration of occlusive compression = OR (12.7), CI		62	Both diagnostic and intervention	Standard Diagnostic and Interventional UFH	Early sheet removal and short time compression with

	() *, P=0.001				protocol	QuikClot® pad can reduce the rate of RAO after trans-radial diagnostic or interventional procedures.
Sharma et al., 2020 [34]	Interruption of blood flow during the hemostasis process p:<0.001		dTRA group: 55 ± 6<br><br>Conventional group: 55 ± 7	**PCI:**78 **CA:**892	200 µg of nitroglycerin and 5000 units of heparin and weight adjusted heparin for the PCI	Dorsal versus classical radial artery vascular access, both procedures have pros and cons. Dorsal overall better from RAO pov but learning techniques and haemostasis devices need to be discovered
Ahmed et al., 2022 [35]	**Early RAO=**Low RAD post FMD OR (0.06), CI (0.01-0.26), p=<0.001<br><br>**Late RAO=** RAD at 1-h post-TR band removal OR (0.09), CI (0.05-0.17), p= <0.001		**62+/-10**	Diagnostic + PCI	200 µg Nitro and verapmil and Heparin 5000 IU	A technique of SURC (simultaneous ulnar radial compression) is associated with less incidence of early and late RAO compared to conventional hemostasis techniques.

Patel et al., 2020 [36]	**Absent patent haemostasis**: OR (137) CI (8-2357) P:<0.01<br><br>**Rebound bleeding:** OR (130) CI (12-1338) P:<0.01	**TR band:**<br><br>**Vasoactive Band:**	**TR band** 57+/-10 **Vasoactive band:** 59+/-10	**PCI:**<br>**CA:**<br>Both	200 µg of nitroglycerin and 2.5 mg of verapamil was administered intra-arterially 5000 IU UFH IV	Ipsilateral ulnar compression performed for the initial 1 hour during the radial hemostatic process after TRA using a dedicated double-balloon device is associated with higher rates of patent hemostasis and lower incidence of RAO compared with a single-balloon band.
Patel et al., 2022 [37]	**Female**= OR (1.18), CI (0.54-2.6), p=0.68<br>**Radil artery diameter**=OR (0.13), CI (0.04-0.38), p=<0.001<br><br>**Large sheath size**=OR (2.9),CI(0.91-9.7), p=0.07<br><br>**Two bladder**	One bladder band= 34 min<br>Two bladder band = 33 min		**CA and PCI**	Heparin 4350 IU given to all	A two-bladder band with simultaneous ipsilateral ulnar artery compression when used for radial artery hemostasis, is associated with a lower incidence of RAO, and can mitigate the penalty for a larger catheter with reassuring implications for use of a 7-Fr

	**band**=OR (0.07), CI (0.01-0.36) p=<0.001<br><br>**Weight=** OR (0.98),CI(0.97-0.99), p=0.002					capable system for complex transradial PCI.
Qin et al., 2022 [38]	**Antiplatelet therapy:** OR (0.41), CI (0.24-0.70), P:0.001<br><br>**RAD:** OR (0.23), CI (0.14-0.37), P:<0.001<br><br>**Haemostasis time:** OR (1.42), CI (1.19-1.69), P:<0.001		61.13 ± 9.51	**Both diagnostic and angioplasty**	**5000 of UFH IV**	Dual-antiplatelet therapy for 1 month after the TRCA was associated with a reduced risk of RAO and deemed safe.
Kanazawa et al., 2022 [39]	**BSA:** OR (1.4), CI (1.19-1.64) P:0.01<br><br>**H/O TRA-PCI** OR (2.35) CI (1.16-5.08) P:0.01	**Conventional Hemostasis** = 13 min<br><br>**Pulse Oxy=** 13.8	**Conventional Hemostasis=** 61.5 ± 9.8<br><br>**Pulse Oxy=** 60.1 ± 11.6	**Diagnostic Angio on all patients**	Standard UFH protocol 0.5 mg isosorbide dinitrate	Oximetry-plethysmography is an efficient and safe method to achieve patent hemostasis after coronary angiography *via* trans-radial access.

Al-Makhamreh et al., 2021 [40]	Repeated use of radial artery		56.9 ± 9.1	**PCI:**57 **CA:**91	500 mcg of nitroglycerin<br>5000 IU UFH<br>During PCI another 3000-5000 IU UFH followed by DAPT	symptoms after catheterization could be related to decreased blood supply of the hand due to occlusion of the blood vessels used for the catheterization.
Achim et al., 2021 [41]	**NAD**	**dTRA-** 41.3 (31.3–51.2)<br>**Control-** 33.2 (30.4–36.1)	66.4 ± 12	**PCI:**966 **CA:**272	Standard heparin dosage	dRA is a reliable and safe vascular access site.
Dwivedi et al., 2022 [11]	**>2Puncture attempts** OR (2.73), CI (2-3.7), P:<0.001<br>**Complex procedure:** OR (3.06), CI (1.9-4.7) P:<0.001<br>**Forearm hematoma** OR (2.5), CI (1.75-3.85),		56.31±10.58	**PCI:**1372 **CA:**380	2.5 mg verapamil and 200 µg nitroglycerin<br>Standard heparin dosage (5000 IU)<br>And adjunct dose in PCI	RAO was not an uncommon complication in trans-radial coronary interventions, especially in the Indian population; and the knowledge of predictors may be helpful in its prevention.<br>Failed to

	P:<0.001<br>**Hemostasis time:**<br>OR (1.004) CI (1.004-1.006), P<0.001					mention association between RAO and dose of heparin
Xie et al., 2021 [42]	Hemostasis techniques		64.6 ± 11.2	**PCI:**363<br><br>**CA:**700	Standard UFH protocol	dTRA is a feasible and safe access and can be used as a rational alternative to traditional radial access for routine coronary interventional procedure.
Voon et al., 2017 [43]	**Air inflation technique for compression technique**		**Sfae guard Radial:** 63.8 ± 10.9<br><br>**TR Band:** 66.8 ± 10.8	**PCI:**84<br>**CA:**0	100-200 mcg nitroglycerin, 250 mcg verapamil and heparin 2000-4000 IU<br><br>For PCI 70-100 IU/kg UFH on top<br><br>dual anti-platelet therapy prior to PCI	Safeguard Radial and TR band did not demonstrate significant between-group differences in short-term RAO incidence
Kherad et al., 2016 [44]	**Gender:**<br>**Age:**<br>**DM: RAD≤**	**NA**	45± 10.8	**PCI:** None<br>**CA:** None	UFH -500IU	the present report shows the feasibility

	**2.5 mm: lower radial peak systolic velocity of ≤ 50 cm/s:**			**For myocardial biopsy**		of using a sheathless catheter to guide access for LV-EMB*. However, duplex sonography revealed a high number of post procedural asymptomatic radial occlusions.
Sanghvi et al., 2018 [45]	**Amount of air time required for patent hemostasi**p: 0.001<br><br>**Radial artery hematoma** p:0.046		**TR group:** 64.34<br><br>**Safeguard group:** 65.21	**PCI:**108<br>**CA:**206	200 µg of Nitroglycerin and 2.5 mg of Verapamil<br><br>UFU heparin as per standard protocol	Evidence-based contemporary TRC protocols of using smaller diameter access, anticoagulation, and use of just enough pressure for the shortest duration of time to achieve hemostasis is associated with very low RAO rate at 30 days irrespective of the radial compression device used.
Santos et al., 2020 [46]	**Peripheral Vascular disease;**		63	**PCI:**104<br>**CA:**496	Standard angiogram and PCI	The incidence of RAO was similar in

	OR(4.99) CI(1.50-16.55) P:0.26				protocol for UHF 75% got dual antiplatelet therapy	patients who received hemostasis with a TR Band versus a pressure dressing after trans-radial cardiac catheterization.
Markovic et al., 2015 [47]	None		67.7 ± 10.2	**PCI:**274 **CA:**95	200 µg of glyceryl trinitrate 5000 U of unfractionated heparin 70 U/kg body weight bolus of UFH for PCI	Radial access coronary catheterization is effective and safe. With a standardized approach, the rates of bleeding events and RAOs are low.
Sanhoury et al., 2022 [48]	**Vascular access site failure:** p<0.001	**dRA:** 24.0 ± 2.91 **Conventional RA:** 22.28 ± 3.83	**dRA:** 56.34 ± 6.08 **Conventional RA:** 57.56 ± 5.49	**PCI:**52 **CA:**48	5 mg verapamil and 5000 IU unfractionated Heparin 100 mg Nitroglycerin	it is a feasible and safe approach for coronary angiography and interventions, especially when using the left d-TRA approach, which has a lower risk of radial artery occlusion. The success rate of d-TRA is proportional to

						the steepness of the operator's learning curve and the quality of the examples chosen
Batchelor et al., 2018 [49]	None	20 mins	**6 Fr Cordis:** 68±10<br>**7 Fr sheathless** **:**64±8.8	**PCI:**41 **CA:**0	Standard heparin protocol	A7Fsheathless approach to TRA-PCI results in no more IMT and early or late RA trauma than a standard 6F sheath/ guide combination, rendering the 7F sheathless technique an attractive option for complex TRA-PCI**.**
Dharma et al., 2018 [50]	**RA compression duration>4 hours** =OR(5.41),CI (231-12.65), p= <0.001	**Nitro group=** 9min<br>**Verapa Group=** 25 mins	**Nitro group=** 56+/-10.8<br>**Verapa group=** 61+/-9.5	**PCI:**677 **CA:**1007	Half patient-verapamil and half nitroglycerine	the use of verapamil or nitroglycerin as a spasmolytic regimen was not associated with RAO.
Eid-Lidt et al., 2022 [51]	**Early RAO-**<br>**hydrophilic introducers** =OR (0.46) CI: (0.27-		60.3 +/-11.1	**PCI:**696 **CA:** 773	5,000 IU of unfractionated heparin (UFH), 2.5 mg verapamil,	the RAO rates at 24 hours, evaluated by plethysmography oximetry and confirmed

	0.76)<br>P = 0.003<br>**intervention al procedures**= OR (0.47) CI (0.27-0.81) P=0.007<br>**Late RAO-** none				and 200 mg nitroglycerin in all patients<br>In PCI 100 iu/kg UFH was added	by ultrasound, of the 3-current radial nonocclusive hemostatic methods (PH, UM, and HD) are not different.
Gaudino et al., 2018 [52]	**Age:** OR (1.00), CI (0.48-2.08), P:0.008		**RA group:** 66+/-9.8 **SV group:** 67.1+/-9.85	**PCI: None CA: None**<br>**CABG**	Standard protocol of UFH	The use of radial-artery grafts resulted in a significantly lower rate of major adverse cardiac events and a better patency rate at a postoperative follow-up of 5 years.
De Sa et al., 2013 [53]	No statistical significance		**Brand new introducer;** 60.1 ± 10.6<br>**Reprocessed Introducers:** 59.4 ± 10.9	**PCI:**65 **CA:**163	5,000 U of heparin, 10 mg of isosorbide mononitrate and 1 mL of lidocaine 2%.<br>IV bolus UFH 100 IU/kg) and added extra 60 IU/kg if	In our study no association was found between reused vascular introducers and early and late RAO in patients undergoing cardiac catheterization.

					procedure > 100 mins POST PCI DAPT	
Gokhroo et al., 2016 [54]	**Tortuosity of vessels** p:0.02	**TUA*:** 17.1+/-1.8<br><br>**TRA:** 16.9+/-1.8	**TUA*:** 67+/-11.4<br><br>**TRA:** 63+/- 12	**PCI:**141 **CA:**1129	50 µg nitroglycerin + 2.5 mg diltiazem + 2 mL 2% lignocaine without preservative	TUA is a viable and non-inferior alternative option for forearm access. In the event of RA cannulation failure, the UA should be considered the second favored crossover site. With increasing experience, TUA may even be considered a default route, thereby sparing the RA for future needs.
Pacchioni et al., 2022 [55]	**Preserved flow:** p;<0.0001<br><br>**Reduced time to hemostasis:** p;<0.0001	**dTRA-** 44+/-33<br><br>**Conventional TRA:** 46+/-33	**dTRA-** 68+/- 11<br><br>**Conventional TRA:** 69+/-11	**PCI:**648 **CA:**515	nitroglycerin 200 mcg and verapamil 5 mg<br><br>5000 IU of unfractionated heparin	DRA was associated with lower rates of RAO compared to TRA. This effect is potentially explained by reduced time-to-hemostasis and maintained flow at the wrist during hemostasis.

Pancholy et al., 2014 [56]	**Lack of UFH** P<0.001	**Warfarin group:** 26 +/- 2<br><br>**Warfarin Group:** 26 +/- 3	**Warfarin Group:** 72+/-9<br><br>**Warfarin Group:** 72+/-11	**PCI:**35 **CA:**301	200 mg of nitroglycerin and 5 mg of diltiazem<br>To all patients<br><br>group 2 received 50 U/kg unfractionated heparin	Patients receiving chronic oral anti-coagulation with warfarin and undergoing transradial coronary angiography without parenteral anticoagulation had a higher incidence of early and late RAO compared with patients receiving standard intravenous heparin therapy.
Lisowska et al., 2015 [24]	**PCI duration** p:0.01	37.2+/- 10	64.0 ± 12.2	**All PCI**	acetylsalicylic acid and clopidogrel) in loading doses. Verapamil 2.5 mg, UHF 5000 IU Extra 300 IU in PCI	The only factor influencing RA patency promptly after the procedure was PCI duration. Moreover, ultrasonographic and clinical control after 6–12 months after the procedure is not necessary because

						persisting obstruction of RA does not generate symptoms.
Kyriakopouloset al., 2021 [57]	**Age:**<br>OR (10.04)<br>CI (1.01-1.07)<br>P:0.006		**Pulse oxy group:**<br>60.1 ± 11.6<br><br>**Control group:**<br>61.5 ± 9.8	**PCI:**102<br>**CA:**197	Standard UFH protocol	Patent hemostasis with the use of pulse oximeter is a simple, efficient, and safe method that is worthy of further investigation
Buturak et al.,2014 [58]	**ratio of sheath/artery diameter (S/A) was > 1=**<br>OR (5.7),<br>CI (3.39–9.80)<br>p= < 0.001<br><br>**post- -procedural access site pain=**<br><br>OR (1.79), CI (1.122–2.861),<br>p=< 0.001	8.4 ± 3.8 min	58.5 ± 9.4	**PCI:**87<br>**CA:**322	500 µg glycerol trinitrate and 2.5 mg verapamil<br><br>Heparin 5,000 IU	Hypertension, post-procedural access site pain and S/A ratio > 1 are independent predictors of RAO at late term.
Monsegu et al, 2012 [59]	**Young radilaist=**<br>p<0.001		**62+/-10**	**PCI:**<br>**CA:**	Vasodilator and heparin standard	TR Band™ device with its flow-limiting

	**No pulse after withdrawn of TR-band=** p<0.001<br>**No use of profile sheath=** p<0.001			**Both**	cocktail (500 μg glycerol trinitrate and 2.5 mg verapamil) Standard UFH	compression allows to reduce early radial artery occlusion as confirmed by DRABAND study, with only 3.8% incidence. We have also shown importance of quality sheath choice avoiding radial occlusion complication.
Dharma et al., 2015 [60]	**Duration of hemostasis:** OR (3.1) CI (1.66-5.82) P:<0.001<br><br>**Lack of nitroglycerine** OR (0.62) CI (0.44-0.87) P:0.006	**20 mins**	59.3+/-10.4	**PCI:**677<br>**CA:**1025	50–100 IU/kg of unfractionated heparin, administered intravenously and intra-arterially.	The administration of nitroglycerin at the **end of** a **trans-radial catheterization**, reduced the incidence of RAO, examined 1 day after the radial procedure by ultrasound. Postprocedural/prehemostasis pharmacologic regimens may represent a novel target for further investigation to reduce RAO

Ognerubvv et al., 2020 [61]	Prolonged hemostasis	**Prolonged group-**23.8 ± 10.8<br><br>**Short group-**23.7 ± 10.1	61.6 ± 9.9	**PCI:**500<br>**CA:**500	Pretreated prior to catheterization with acetylsalicylic acid (125 mg daily) and clopidogrel (75 mg daily)	Shorter hemostasis was associated with significantly less RAO compared to prolonged hemostasis
Hahalis et al.,<br>2018 [18]	**Male:**<br>OR (0.63)<br>CI (0.45-0.88)<br>P:0.007<br><br>**Arterial spasm:**<br>OR (2.59)<br>CI (1.55-4.33)<br>P:<0.001<br><br>**Number of attempts:**<br>OR (1.19)<br>CI (1.08-1.30)<br>P:<0.001<br><br>**Low dose Heparin:**<br>OR (0.20)<br>CI (0.12-0.34)<br>P:<0.001	**8+/-5**	**Low Heparin Group:**<br><br>64.1+/-11.7<br><br>**High heparin group**:<br><br>64.7+/-11.3	**PCI:0**<br>**CA:**1836	Low and high dose heparin	High compared with standard heparin dose significantly reduced the rate of RAO in patients<br><br>undergoing coronary angiography. High-intensity anticoagulation should be considered in trans-radial diagnostic procedures

Sadaka et al., 2019 [20]	**Female:** p<0.001<br><br>**Age:** p<0.001<br><br>**Compression duration:** p<0.001<br><br>**RAD:** p<0.001	24.43 ± 26.36	57.7 ± 8.8	**PCI:164**<br>**CA:0**	5000 IU (UFH)<br><br>nitroglycerine, verapamil,<br><br>And added dose of UFH for PCI	RAO, although clinically a silent issue, has been the main complication following TRA. In patients with high predictors of RAO, careful management and close follow-up are required to ensure radial artery long-term patency.
Jirous et al., 2020 [62]	**NAD**	12+/10	65 ± 9.4	**PCI:**121<br>**CA:**379	5000 units of UFH	RBT has the potential to be used as the first method of detection of radial occlusion after coronary catheterizations.
Campos et al, 2018 [63]	**NAD**	**G30:** 22.9±9.9<br>**G60:** 23.5±9.3	**G30:** 60<br>**G60:** 62	**PCI:303**<br>**CA:0**	50U/kg of UFH	The different compression times, either 30 or 60 minutes, applied on the radial artery after trans radial coronary angiography did not significantly

						influence the occurrence of hemostasis and immediate complications

Supplemental Table S3. Risk-of-bias assessment for randomized trials (RoB 2).

Study Name	Outcome	Bias arising from the randomization process	Bias due to deviations from intended interventions	Bias due to missing outcome data	Bias in measurement of the outcome	Bias in selection of the reported result	Overall risk of bias
Liang 2022	RAO	🟢	🟢	🟢	🟢	🟢	🟢
Ahmed 2022	RAO	🟢	🟢	🟢	🟢	🟢	🟢
Eid-Lidt 2022	RAO	🟢	🟢	🟢	🟢	🟢	🟢
Kyriakopoulos 2021	RAO	🟢	🟢	🟢	🟢	🟢	🟢
Sharma 2020	RAO	🟡	🟢	🟡	🟡	🟢	🟡
Ognerubov 2020	RAO	🟢	🟢	🟢	🟢	🟢	🟢
Patel 2020	RAO	🟢	Unclear	🟢	🟢	🟢	🟢
Santos 2020	RAO	🟢	🟡	🟡	🟢	🟢	🟢
Dharma 2018	RAO	Unclear	🟢	🟢	Unclear	🟢	Unclear
Hahalis 2018	RAO	🟢	🟢	🟢	🟢	🟢	🟢
Batchelor 2018	RAO	🟢	🟢	🟢	🟢	🟢	🟢
Shangvi 2018	RAO	🟢	🟢	🟢	🟢	🟢	🟢

Study Name	Outcome	Bias arising from the randomization process	Bias due to deviations from intended interventions	Bias due to missing outcome data	Bias in measurement of the outcome	Bias in selection of the reported result	Overall risk of bias
Campos 2018	RAO	●	●	●	●	●	●
Gaudino et al., 2018		●	●	●	●	●	●
Voon 2017	RAO	●	●	●	●	●	●
Aykan 2015	RAO	●	●	●	●	●	●

● = Low Risk of Bias

● = Some Concerns of Bias

● = High Risk of Bias

Supplemental Table S4. NEWCASTLE - OTTAWA Quality Assessment for Observational Studies .

Publication year	Study Type	Selection	Comparability	Outcome	Total Score
Roy et al., 2021	Case Series				
Rammos et al., 2018	Case Control	****	**	***	9
Schlosser et al., 2022	Prospective Cohort	*	**	**	5
Aminian, et al., 2014	Retrospective	***	*	**	6
Patel et al., 2022	Retrospective	****	0	***	7
Qin et al., 2022	Prospective study	**	0	***	5
Kanazawa et al., 2022	Retrospective study	**	*	***	6
Al-Makhamreh et al., 2021	Retrospective study	*	0	***	4
Achim et al., 2021	Prospective Study	***	**	***	8
Dwivedi et al., 2022	Prospective study	**	*	**	4
Xie et al., 2021	Retrospective study	***	0	**	5
Kherad et al., 2016	Prospective Study	**	0	**	4
Markovic et al., 2015	Prospective study	***	*	***	7
Dharma et al., 2018	Post hoc analysis of RCT	**	*	**	5
Gaudino et al.,	Prospective				

2018	cohort study				
Gokhroo et al., 2016	Observational study	**	*	***	**6**
Pacchioni et al., 2022	Propensity matching study	**	**	***	**7**
Pancholy et al., 2014	Retrospective study	**	**	**	**6**
Lisowska et al., 2015	Prospective cohort study	***	**0**	**	**5**
Buturak et al.,2014	Prospective cohort study	***	*	**	**6**
Monsegu et al, 2012	Prospective cohort study				
Sadaka et al, 2019	Prospective cohort study	***	**0**	***	**6**
Jirous et al, 2020	Prospective cohort study	***	**	***	**8**

Assessment Scores:

7-9 = High quality

4-6= High risk of bias

0-3= Very High risk of bias

Supplemental Table S5. Meta-Regression Analysis (Early vs Late RAO)

Early RAO

Variable	Coefficient	Standard error	Z score	*p* value	95% CI	
Percutaneous intervention	0.0000202	0.0000489	0.41	0.679	-0.0000757	0.0001161
Coronary angiography	-0.0000207	0.0000299	-0.69	0.488	-0.0000793	0.0000379
Catheter size	0.0437724	0.0329469	1.33	0.184	-0.0208025	0.1083472
age*	0.000357	0.0033857	-2.76	0.006	-0.0159928	0.0027212

Late RAO

Variable	Coefficient	Standard error	Z score	*p* value	95% CI	
Percutaneous intervention	-0.0001173	0.0001673	-0.70	0.483	-0.0004453	0.0002107
Coronary angiography	-0.0000231	0.0001663	-0.14	0.889	-0.000349	0.0003028
Catheter size	-0.0161084	0.086979	-0.19	0.853	-0.1865841	0.1543673
age	0.4735354	0.9196591	0.51	0.607	-1.328963	2.276034
